# Genome-Wide Characterization of the F-Box Gene Family in *Cardamine hupingshanensis* and Functional Analysis of *ChFBX171*

**DOI:** 10.3390/biology15131003

**Published:** 2026-06-25

**Authors:** Yifan Wang, Yan Yu, Xiaorong Xiao, Qiaoyu Tang, Zhixin Xiang, Shengcai Chen, Zhi Hou, Yifeng Zhou, Yanke Lu

**Affiliations:** 1 Hubei Key Laboratory of Biological Resources Protection and Utilization, Hubei Minzu University, Enshi 445000, China; 2 College of Biological and Food Engineering, Hubei Minzu University, Enshi 445000, China; 3 Cereal Crops Institute, Hainan Academy of Agricultural Sciences, Haikou 571100, China; 4 College of Forestry and Horticulture, Hubei Minzu University, Enshi 445000, China; 5 Sanya Institute of Henan University, Henan University, Sanya 572000, China

**Keywords:** *Cardamine hupingshanensis*, F-box, abiotic stress, *ChFBX171*, salt stress

## Abstract

*Cardamine hupingshanensis* (*C. hupingshanensis*) is an important dietary source of selenium for humans due to its remarkable capacity for selenium hyperaccumulation. As core components of the SCF (SKP1–Cullin–F-box) ubiquitin ligase complex, F-box proteins play vital roles in plant responses to environmental stress, such as salt and drought. However, information regarding the F-box gene family in *C. hupingshanensis* and its potential functions in regulating responses to abiotic stress remains limited. In this study, members of the F-box gene family in *C. hupingshanensis* were identified through sequence alignment. Comprehensive bioinformatic analyses, including analyses of physicochemical properties, phylogenetic relationships, subcellular localization, conserved motifs and domains, gene structure, chromosomal distribution, promoter *cis*-elements, and gene duplication events, were performed using TBtools and associated online resources. In particular, a total of 548 F-box genes were identified and classified into nine distinct groups based on phylogenetic analysis. Protein sequence analysis predicted 15 conserved motifs and 18 distinct domains across the identified F-box proteins. Promoter analysis suggested the presence of 32 different *cis*-elements that may be potentially associated with growth, development, hormone signaling, and abiotic stress responses. Furthermore, 283 collinear gene pairs were detected within the *C. hupingshanensis* genome, providing insights into the possible expansion of this gene family. Quantitative real-time PCR was employed to examine the tissue-specific expression levels of F-box genes in various organs, as well as their expression profiles in response to exogenous selenium, salt, osmotic stress, and abscisic acid treatment. The results indicated that 11 *ChFBX* genes responded to exogenous selenium, salt, osmotic stress, or abscisic acid. Notably, transgenic plants overexpressing *ChFBX171* displayed heightened sensitivity to salt stress during seed germination. In conclusion, this study provides a comprehensive identification and characterization of 548 F-box genes in *C. hupingshanensis* and offers valuable insights into the potential role of *ChFBX* genes, particularly *ChFBX171*, in mediating responses to abiotic stress.

## Simple Summary

Plants often struggle to grow when exposed to abiotic stresses, which can significantly reduce crop yields. In this study, we identified 548 F-box genes from *Cardamine hupingshanensis*, a unique plant that naturally accumulates high levels of selenium. We performed functional analysis and expression profiling of representative F-box genes in response to selenium, salt, osmotic stress, and abscisic acid (ABA) treatment. Notably, the research found that *ChFBX171* increases plant sensitivity to salt during seed germination. This study provides a valuable genomic resource and suggests potential associations of certain *ChFBX* genes with abiotic stress responses.

## 1. Introduction

Protein ubiquitination is a critical post-translational modification involving the covalent attachment of ubiquitin to target proteins via an E1-E2-E3 enzymatic cascade, thereby regulating their degradation, localization, or activity [1]. Within this cascade, E3 ubiquitin ligases play a central role in determining substrate specificity. Based on structural and functional characteristics, E3 ligases are primarily classified into three types: RING, HECT, and RBR. Among RING-type E3s, the SCF (Skp1–Cullin1–F-box protein) complex is one of the most extensively characterized [2]. As a key component of the SCF complex, F-box proteins mediate the selective degradation of target proteins through the ubiquitin–proteasome pathway [3]. This degradation system is essential for maintaining protein homeostasis and regulating various physiological processes, including cell cycle progression and stress responses [4,5,6]. Through targeted protein degradation, these regulators enable plants to rapidly adjust their physiology and gene expression to enhance survival.

In plants, F-box proteins have been increasingly implicated in responses to various abiotic stresses. Among them, salt stress is one of the major limiting factors affecting plant growth and crop productivity worldwide. Accumulating evidence has highlighted the essential roles of F-box proteins in mediating salt tolerance through diverse regulatory mechanisms. For instance, in Arabidopsis, the F-box protein EST1 modulates salt tolerance by regulating plasma membrane Na(+)/H(+) antiport activity [7]. The SCF E3 ligase PP2-B11 also plays a positive role in response to salt stress in Arabidopsis [8]. Similarly, TaFBA-2A improved the salt tolerance and increased the JA responsiveness of the transgenic rice lines in wheat [9]. In soybean, overexpression of the *GmFBX193* gene reduced salt sensitivity in Arabidopsis and affected the transcription levels of some stress-responsive genes [10]. Furthermore, F-box proteins have been shown to directly interact with and target key negative regulators of salt stress for degradation, such as components of the SOS (SALT OVERLY SENSITIVE) pathway or ion transporters, thus maintaining cellular ion homeostasis under salt stress [11]. In addition to the above functions, recent studies have also implicated F-box proteins in metal element absorption [12,13,14]. As key determinants of substrate specificity in the ubiquitin–proteasome system, F-box proteins have undergone remarkable expansion in plants, with 694 members identified in the model plant *Arabidopsis thaliana* [15] and 687, 359, 517, 509, 592, 972, and 139 members in rice (*Oryza sativa*) [16], maize (*Zea mays*) [17], soybeans (*Glycine max*) [18], apples (*Malus domestica*) [19], cotton (*Gossypium hirsutum*) [20], *Medicago truncatula* [21] and tomatoes (*Solanum lycopersicum*) [22].

*C. hupingshanensis*, which is also known as *C. violifolia* or *C. enshiensis*, is a novel selenium hyperaccumulator plant native to the Wuling Mountain area of China. It was initially discovered by resource development scientists in the selenium-mining trenches of Yutangba, Enshi City, the site of the world’s highest grade of selenium ore, and concurrently identified by taxonomists in the Hupingshan National Nature Reserve of Hunan Province [23]. Studies have shown that *C. hupingshanensis* grows well under high-selenium conditions, efficiently absorbing inorganic selenium and converting it into organic forms such as selenocystine and selenomethionine. The selenium concentration in its leaves reaches up to 1427 mg kg^−1^ [24]. This remarkable property makes *C. hupingshanensis* an ideal model for investigating selenium metabolism, hyperaccumulation, and detoxification in plants.

The human body mainly acquires Se from plant foods, including staple cereals such as rice and wheat, as well as various vegetables. In this study, *C. hupingshanensis*, a vegetable known as the “king of Se-enriched plants,” was used as the experimental material. This species exhibits a strong ability to accumulate selenium from the environment [22]. In 2021, the genome of *C. hupingshanensis* was sequenced, which promoted research on the genomics of this plant [25]. Although there are numerous members in the F-box family, reports on F-box involvement in selenium accumulation and abiotic stress regulation are scarce in *C. hupingshanensis*. Here, 548 F-box genes were identified in *C. hupingshanensis* and the detailed analyses of their structures, chromosomal distributions, evolutionary relationships, protein-coding structures, and physicochemical properties were performed. In addition, the transcriptional expression patterns of these genes in response to exogenous selenium as well as salt, osmotic, and ABA stress were also analyzed. Interestingly, Arabidopsis overexpressing *ChFBX171* exhibited increased sensitivity to salt stress during the seed germination stage, suggesting that *ChFBX171* may be involved in regulating salt stress signaling in *C. hupingshanensis*. These findings provide a comprehensive genomic resource for the F-box gene family in *C. hupingshanensis* and offer a preliminary functional characterization of *ChFBX171*, suggesting its possible involvement in salt stress adaptation.

## 2. Materials and Methods

### 2.1. Plant Materials and Growth Conditions

The wild-type *C. hupingshanensis* variety sourced from the Yutangba Mine in Enshi, Hubei Province, China (109°46′39″ E and 30°20′16″ N); *Arabidopsis thaliana* Col-0; and transgenic Arabidopsis lines overexpressing *ChFBX171* were used in this study.

For tissue expression analysis, the seeds were germinated in distilled water for 7–10 days until two cotyledons had developed. Then the germinated seedlings were transferred to a sandy substrate for cultivation under a 12 h light/12 h dark photoperiod with light at a photon density of about 50 µmol m^−2^ s^−1^ supplied by light bulbs at 22 °C in the plant culture chamber. The *C. hupingshanensis* samples were cultivated for 5–6 months in a sandy substrate until flowering, after which roots, stems, leaves, and flowers were collected for RNA extraction.

To study the effects of different abiotic stress factors on the expression of F-box genes, the germinated seedlings were transplanted into pots and cultivated hydroponically with the Hoagland nutrient solution (Coolaber, Beijing, China) under a 12 h light/12 h dark photoperiod with light at a photon density of about 50 µmol m^−2^ s^−1^, which was replaced every seven days, until the plants developed 6–7 true leaves. The plants were then treated with Hoagland nutrient solutions containing 0.25 mg/L or 16 mg/L Na_2_SeO_4_ (Sigma-Aldrich, Darmstadt, Germany) [26], 150 mM NaCl (Sinopharm Chemical Reagent Co., Ltd., Shanghai, China), 20% PEG6000 (Sinopharm Chemical Reagent Co., Ltd., Shanghai, China)or 50 μM ABA (Coolaber, Beijing, China) for 0, 3, 6, 12 h, respectively. Each treatment was set up with 3 biological replicates. Leaves and roots were sampled, rapidly frozen in liquid nitrogen and stored at −80 °C.

For the germination experiment, seeds of the wild-type and overexpression Arabidopsis lines harvested from the same batch were sown on the 1/2 MS solid medium without or with 150 mM NaCl, respectively. Twenty-five seeds were used per replicate, and two biological replicates were cultivated per experiment. The experiment was conducted at least twice independently. The seeds were then stratified at 4 °C for 3 days, followed by transfer to a growth chamber for germination. Germination was scored after 24 h and subsequently every 24 h to calculate the germination rate.

### 2.2. Genome-Wide Identification of F-Box Genes in C. hupingshanensis

The whole-genome files of *C. hupingshanensis*, including the gene annotation GTF file, the nucleotide sequence FASTA file and the protein sequence FASTA file, were downloaded from the Genome Warehouse Big Data Center (accession number PRJCA005533), and the protein sequences of 694 *Arabidopsis thaliana* F-box genes were retrieved from the TAIR website (https://www.arabidopsis.org/, accessed on 11 October 2024). To identify putative F-box gene members in *C. hupingshanensis*, a multi-step filtering pipeline was established. First, BLASTP searches were performed using the BLAST Zone tool in TBtools (version 2.0), with the 694 *A. thaliana* F-box protein sequences as queries against the *C. hupingshanensis* proteome using thresholds of E-value ≤ 1 × 10^−5^, identity ≥ 30%, and query coverage ≥ 50%. The Fasta Extract tool in TBtools was subsequently used to retrieve the protein sequences of the candidate F-box genes. Candidate sequences with lengths below 100 amino acids were discarded, and for multiple transcripts originating from the same gene locus, only the representative sequence with the highest bit score was retained. All remaining candidates were subjected to domain integrity validation using InterProScan (InterProScan—InterPro, https://www.ebi.ac.uk/interpro/search/sequence/, accessed on 23 October 2024) which integrates HMM-based searches against the Pfam database (InterPro, https://www.ebi.ac.uk/interpro/, accessed on 3 November 2024), in conjunction with the NCBI Conserved Domain Database (Home—Conserved Domains—NCBI, https://www.ncbi.nlm.nih.gov/cdd/, accessed on 17 November 2024), with parameters of E-value ≤ 1 × 10^−5^ and domain coverage ≥ 50%. Additionally, the protein sequences were further verified using Protein BLAST on the NCBI BLAST website (https://blast.ncbi.nlm.nih.gov, accessed on 1 December 2024) to ensure their accuracy and authenticity. Only sequences containing a complete and conserved F-box domain were retained as high-confidence ChFBX members.

### 2.3. Chromosomal Localization and Phylogenetic Analysis of ChFBX Genes

The chromosomal localization information of *ChFBX* genes was extracted from the whole-genome annotation file of *C. hupingshanensis* and subsequently visualized using the “Gene Location Visualize from GTF/GFF” tool in TBtools software. The protein sequences of ChFBX genes were extracted using TBtools software and aligned using ClustalW (https://www.genome.jp/tools-bin/clustalw, accessed on 11 December 2024) with default parameters (gap opening penalty = 10, gap extension penalty = 0.2, and protein weight matrix = Gonnet). The alignment was manually inspected to ensure reasonable quality, and no additional gap trimming was performed. The best-fitting amino acid substitution model was determined using the Model Selection function in MEGA 11 based on the Bayesian Information Criterion (BIC), and the JTT + G + F model was selected. A maximum likelihood (ML) phylogenetic tree was constructed using MEGA 11 (https://www.megasoftware.net/, accessed on 4 January 2025) with the JTT + G + F model, 1000 bootstrap replicates, and complete deletion of gaps. Further optimization of the phylogenetic tree was performed using FigTree software (version 1.4.3), including adjustments to font styles and branch colors, to obtain a more visually enhanced phylogenetic tree for an in-depth analysis of the evolutionary relationships among *ChFBX* genes. Based on the phylogenetic topology (monophyletic clades with bootstrap support ≥ 50%) and conserved C-terminal domain architectures (e.g., FBA, FBD, LRR, Kelch, etc.), the 548 ChFBX proteins were classified into distinct subfamilies.

### 2.4. Physicochemical Properties and Domain Analysis of ChFBX Genes

The protein sequences of *ChFBX* genes were individually submitted to ExPASy (https://web.expasy.org/protparam/, accessed on 21 January 2025) to obtain basic physicochemical properties for each gene, including molecular weight (kDa), amino acid count (aa), isoelectric point (pI), hydropathicity (GRAVY), and instability index. Meanwhile, their protein sequences were submitted as a FASTA file to WoLF PSORT (https://wolfpsort.hgc.jp/, accessed on 1 February 2025), with the species set to plants, to predict their subcellular localization. The conserved domains of ChFBX genes were identified using the Batch CD-search tool on the NCBI website (National Center for Biotechnology Information, https://www.ncbi.nlm.nih.gov/, accessed on 13 February 2025), with the results saved as a “hitdata.txt” file. The ChFBX protein sequences were submitted to the MEME website to identify conserved motifs, with the maximum motif count set to 15. The results were saved in “XML” format. The annotation file of FBX genes was extracted using the “GXF select” tool in TBtools software and saved in “GFF” format. Finally, the phylogenetic tree (“NWK” file), conserved motifs (“XML” file), gene annotation (“GFF” file), protein sequences (“FASTA” file), and conserved domain (“hitdata” file) were visualized using the “Gene Structure View (Advanced)” tool in TBtools. No parameters were altered from their default settings. The resulting image was further refined using Adobe Illustrator 2021 for enhanced presentation.

### 2.5. Promoter Cis-Elements and Collinearity Relationship Analysis of ChFBX Genes

Using TBtools software, the 2 kb upstream promoter sequences of *ChFBX* genes were extracted from the whole-genome sequence of *C. hupingshanensis* and submitted to the PlantCARE website (http://bioinformatics.psb.ugent.be/webtools/plantcare/html, accessed on 4 March 2025) for *cis*-element analysis. The Basic Biosequence View tool in TBtools software was used to visualize the *cis*-elements of *ChFBX* genes. The intra-species genomic collinearity of *C. hupingshanensis* and its inter-species collinearity with *Arabidopsis thaliana* and *Oryza sativa* were analyzed using the “One Step MCScanX-Super Fast” tool in TBtools (version 2.0) with default parameters (E-value = 1 × 10^−10^, num_hits = 5). This tool integrates BLASTP search with the MCScanX algorithm to generate the corresponding collinearity files. The “Advanced Circos” tool in TBtools was then employed to visualize the intra-species collinearity of *ChFBX* genes for subsequent evolutionary relationship analysis. Additionally, the “Dual Synteny Plot” tool was used to visualize the inter-species collinearity of *ChFBX* genes between *C. hupingshanensis* and *A. thaliana* or *O. sativa*.

### 2.6. GO and KEGG Enrichment Analyses of ChFBX Genes

The ChFBX protein sequences were submitted to the eggNOG-mapper website (http://eggnog6.embl.de/app/phyloprofile/, accessed on 8 May 2025) for Gene Ontology (GO) and KEGG enrichment analyses. The resulting files were preliminarily analyzed using the “eggNOG-mapper Helper” tool in TBtools. Subsequently, the “GO” and “KEGG” files were subjected to GO and KEGG pathway enrichment analyses respectively using the “GO Enrichment” and “KEGG Enrichment Analysis” tools. Finally, the results of the GO and KEGG pathway enrichment analyses were visualized using the “Enrichment Bar Plot” tool.

### 2.7. Total RNA Extraction and qRT-PCR

For gene expression analysis, total RNA was extracted using the TransZol Up Plus RNA Kit (TransGen Biotech, Beijing, China) and reverse-transcripted to cDNA using a reverse transcription kit from TaKaRa according to the manufacturer’s instructions (TaKaRa, Dalian, China). Subsequently, the expression of target genes in the samples was detected using the Hief qPCR SYBR Green Master MixKit (Yeasen Biotech, Shanghai, China). Each reaction mixture (10 μL) consisted of 5 μL of the Hief qPCR SYBR Green Master Mix (High Rox Plus), 1 μL of cDNA, 0.2 μL of the forward primer (10 μmol L^−1^), 0.2 μL of the reverse primer (10 μmol L^−1^), and 3.6 μL of RNase-free ddH_2_O. *ChActin* was employed as the reference gene, and all reactions were run in three technical replicates. Real-time qPCR was performed on an ABI StepOnePlus apparatus (Thermo Fisher Scientific, Waltham, MA, USA), and gene expression was calculated using the 2^−ΔΔCt^ method. For all qRT-PCR reactions, melt curve analysis was performed after each run to verify the specificity of amplification. The average Ct values from the 3 technical replicates were used for further analysis. The statistical significance of expression differences was assessed using Student’s *t*-test, as appropriate, with a significance threshold of *p* < 0.05. The results were analyzed and visualized using R (version 4.0). The primers used for real-time qPCR analysis are listed in Table S1.

### 2.8. RNA-Seq Data Analysis and Screening of Candidate F-Box Genes

Raw RNA-seq data were retrieved from NCBI under the accession number PRJNA590869. Adapters and low-quality bases (q < 30) were trimmed using Trim Galore (version 2.2.0). Clean reads were then mapped to the *C. hupingshanensis* reference genome using HISAT2 (version 2.2.1) with the parameters “no-mixed and no-discordant”. Reads mapping to the exons of each gene were counted by FeatureCounts (version 2.0.6). The overall mapping rates of the samples were over 90% (Control: 94.51%, 95.07%, and 93.97%; 0.25 mg/L: 94.73%, 95.24%, and 94.54%; 16 mg/L: 93.86%, 94.16%, and 94.69%). Gene expression levels were normalized using the FPKM (Fragments Per Kilobase of transcript per Million mapped reads) method. Principal component analysis (PCA) was performed using the R package “factoextra” based on the normalized FPKM values to evaluate consistency among biological replicates. Differentially expressed genes (DEGs) were identified using DESeq2 (version 1.38.0) based on raw counts, with the criteria of |log_2_FC| > 1 and FDR < 0.05. Among the DEGs, F-box family members were extracted based on keyword annotation. Candidate F-box genes with FPKM (Fragments Per Kilobase of transcript per Million mapped reads) values > 10 were considered highly expressed. After applying these criteria to both selenium treatment groups, the resulting gene lists were merged, yielding 11 candidate F-box genes for further gene expression analysis.

### 2.9. Plant Transformation and Semi-Quantitative RT-PCR

The *ChFBX171* coding sequence was cloned into a 35S-driven overexpression vector and introduced into Agrobacterium tumefaciens GV3101. *Arabidopsis thaliana* Col-0 plants were transformed using the floral dip method. Transgenic plants were selected on the 1/2 MS medium containing 50 mg/L hygromycin. For semi-quantitative RT-PCR, total RNA was extracted from T3 homozygous lines using the TransZol Up Plus RNA Kit (TransGen Biotech, Beijing, China). After reverse transcription, PCR was performed using gene-specific primers for *ChFBX171*, with Arabidopsis *Actin2* as an internal control. PCR products were resolved on 1.2% agarose gels and visualized under UV light. Three overexpression lines (OE6, OE9, and OE11) with elevated *ChFBX171* expression levels were selected for further experiments. The primers used for semi-quantitative real-time qPCR analysis are listed in Table S1.

### 2.10. Statistical Analysis

The expression levels of *ChFBX* genes under NaCl, PEG6000, and ABA treatments were statistically analyzed using two-way analysis of variance (treatment × time). A significant treatment-by-time interaction was detected, prompting subsequent simple effect analyses. At each time point, differences between each treatment group and the corresponding control (CK) were assessed using Student’s *t*-test. To correct for multiple comparisons, the Bonferroni method was applied, and the significance threshold was adjusted to (* *p* < 0.05/*n*) [27], where *n* denotes the number of comparisons within each treatment condition. All statistical analyses were carried out using R software (version 4.0), with three biological replicates per dataset. Bar charts were generated in R (version 4.0) and then refined and assembled using Adobe Illustrator 2023 software.

## 3. Results

### 3.1. Identification and Physicochemical Property Analysis of ChFBX Genes

**Figure 1:**
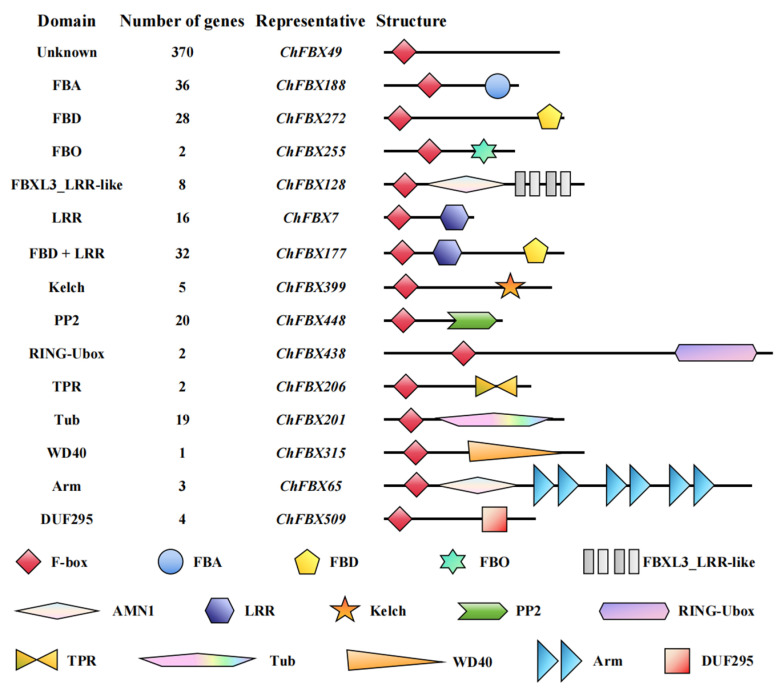
Domain analysis of the 548 F-box proteins in *C. hupingshanensis*.

To identify gene members of the F-box family in *C. hupingshanensis*, the whole-genome and relative files of *C. hupingshanensis* were first downloaded from the Genome Warehouse Big Data Center. Then the amino acid sequences of 694 *Arabidopsis thaliana* F-box proteins were used as queries to perform a blast search in TBtools software, resulting in 1100 putative F-box proteins. By domain analysis, 548 proteins were identified to contain F-box domains and named as *ChFBX1-ChFBX548* according to their location on the chromosomes of *C. hupingshanensis* (Table S2). The specific domains of each F-box protein were also analyzed according to the conserved domains at the C-terminal (Figure 1). As shown in Figure 1, most F-box proteins (370) have unknown domains in the C-terminal. The other F-box proteins have special domains, which include FBA (36), FBD (28), FBO (2), FBXL3_LRR-like (8), LRR (16), FBD and LRR (32), Kelch (5), PP2 (20), RING-Ubox (2), TPR (2), Tub (19), WD40 (1), Arm (3) and DUF 295 (4).

The basic physicochemical properties of 548 ChFBX genes were analyzed (Table S3), including amino acid length, isoelectric point (PI), protein molecular weight (MW), protein instability index, and grand average of hydropathicity (GRAVY), and the subcellular localization of these proteins was also predicted. The results showed significant variability in each of the physicochemical properties among these F-box proteins. The encoded protein lengths of these genes ranged from 63 to 1318 amino acids, with predicted molecular weights ranging from 7.10 to 148.56 kDa. The isoelectric point analysis result showed that 234 F-box proteins were acidic proteins, with a minimum PI of 4.41. A total of 314 F-box proteins were basic proteins, with a maximum PI of 10.09. The protein instability index is an important indicator of protein stability and the relative analysis result showed that the instability index of 431 F-box proteins was >40, which indicated that most F-box proteins were unstable. As F-box proteins are involved in regulating multiple biological processes, including the regulation of growth and development, as well as responses to environmental stress, this instability may be related to their role in dynamically regulating target proteins. The grand average of hydropathicity of 435 F-box proteins was below zero, suggesting a hydrophilic nature. The subcellular localization of these F-box proteins was also predicted by WoLF PSORT (Figure S1 and Table S3). The result showed that 220 F-box proteins were localized in the nucleus, 174 F-box proteins were localized in the cytoplasm, 110 F-box proteins were localized in the chloroplast, 12 F-box proteins were localized in the mitochondrion, and the other F-box proteins were localized in the plasma membrane, peroxisome, Golgi apparatus and other organelles. These results indicated that most of the F-box proteins may function in the nucleus, cytoplasm, chloroplast and mitochondrion, and a few of them may function in other organelles.

### 3.2. Phylogenetic Tree and Domain Analysis of ChFBX Genes

**Figure 2:**
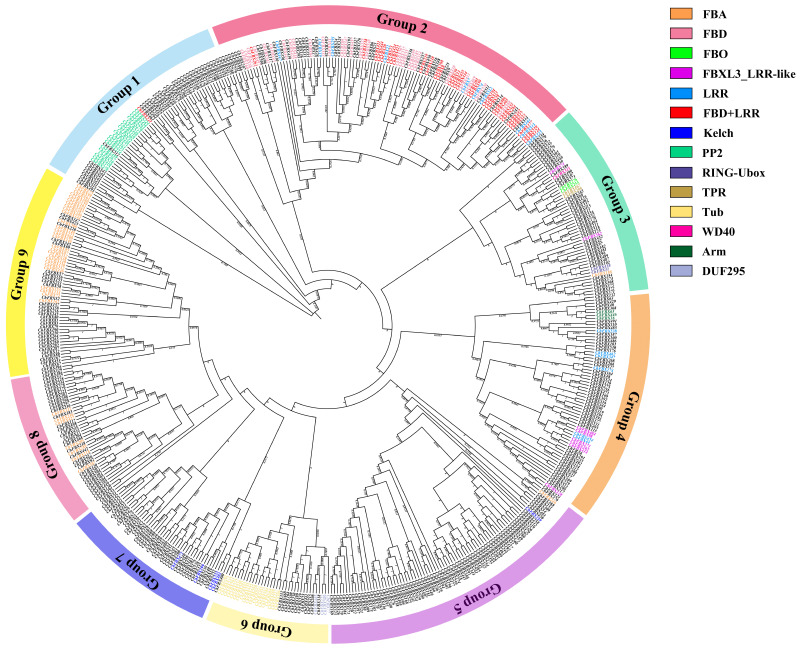
Phylogenetic tree of the F-box proteins in *C. hupingshanensis*. The F-box proteins were grouped into 9 distinct subgroups. Each group in the phylogenetic tree is represented by a distinctive color. Proteins containing different conserved domains are marked with their corresponding colors.

A comparative analysis and identification of the 548 F-box protein sequences were performed using MEGA11 software and the phylogenetic tree was constructed using the maximum likelihood method, clearly outlining the evolutionary relationships among these genes. Based on the phylogenetic relationships, 548 F-box proteins were divided into nine distinct groups (Figure 2). In detail, there were 59 proteins in group 1 that mainly contained the PP2 domain. Group 2 contained 106 proteins that mainly contained the FBD and LRR domains. Group 6 contained 35 proteins, which mainly possess the Tub domain. A total of 60 proteins in group 9 mainly contained the FBA domain. A total of 54, 66, 80, 45 and 43 proteins were in group 3, group 4, group 5, group 7 and group 8 without many specialized domains. These results showed that the proteins in the same subfamily frequently contained the same domain, suggesting similar biological functions of these F-box proteins.

**Figure 3:**
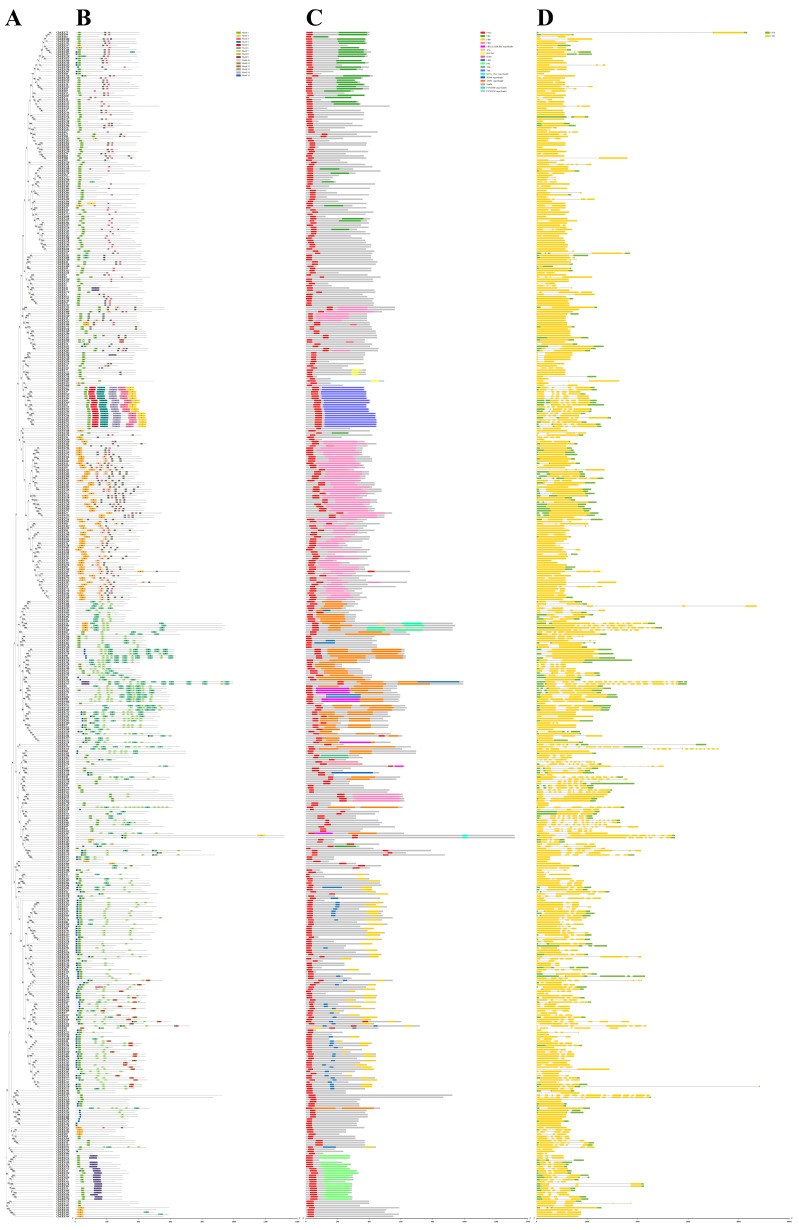
Phylogenetic tree, conserved motifs, structural domains, and gene structures of F-box genes in *C. hupingshanensis*. (**A**) The phylogenetic tree of 548 ChFBX genes. (**B**,**C**) The conserved motifs and structural domains of 548 ChFBX genes; different conserved motifs and structural domains are marked by different colors. The color legend is in the upper right corner in each figure. (**D**) Intron and exon structures of 548 *ChFBX* genes; exons and untranslated regions (UTRs) are represented by yellow and green boxes, while introns are represented by gray lines.

Phylogenetic relationships, conserved motifs, conserved domains, and gene structure were determined on the complete sequences of 548 F-box proteins (Figure 3). Fifteen different motifs were identified in these amino acid sequences and motif 1 was present in most of the F-box protein sequences. Eighteen distinct domains were identified in all F-box proteins. In addition to the conserved F-box domain, these proteins harbor other characteristic domains such as FBA, FBD, FBO, Arm, Kelch, LRR, and WD40 repeats, indicating their conserved role and potential importance in regulatory functions. We also found that the F-box proteins in the same phylogenetic branch shared similar types and numbers of motifs and domains. This similarity may be because genes with similar functions tend to have similar conserved motifs, which remain relatively stable throughout evolution and are preserved by natural selection. The F-box gene structures including the length and number of exons, introns and untranslated regions (UTRs) were also analyzed. The results showed that genes within the same branch of the phylogenetic tree for each subfamily generally shared similar structures, while genes on more distantly related branches displayed dissimilar structures. These differences were reflected in the distribution of their exons, introns and untranslated regions. This might be due to the various functions of the proteins encoded by these genes or the presence of regulatory elements that influence gene expression levels within the untranslated regions.

### 3.3. Chromosomal Distribution and Duplication Events of ChFBX Genes

**Figure 4:**
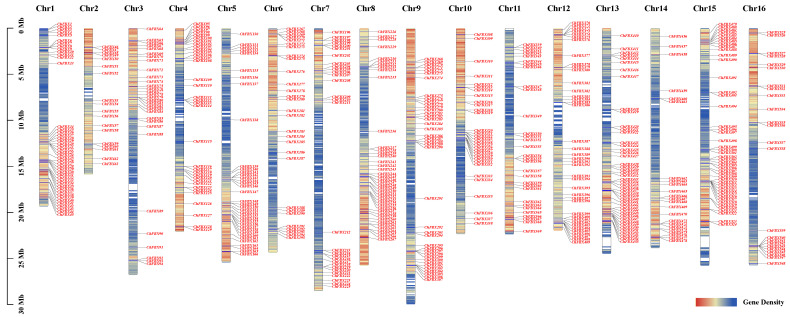
Chromosomal localizations of F-box genes on 16 chromosomes of *C. hupingshanensis*. The colors of the chromosomes indicate gene density.

Using the TBtools software for visualization, it can be observed that 548 F-box genes are unevenly distributed across the 16 chromosomes of *C. hupingshanensis* (Figure 4). Each chromosome contained a different number of F-box genes. Chr15 and Chr13 contain the highest number of F-box genes in the genome (46 each, 16.79%), followed by Chr1 (45, 8.21%), Chr8 (42, 7.66%), Chr9 (40, 7.30%), and Chr12 (40, 7.30%). Conversely, Chr5 (37, 6.75%), Chr4 (35, 6.39%), Chr3 (31, 5.66%), Chr10 (31, 5.66%), Chr11 (31, 5.66%), and Chr7 (30, 5.47%) contained between 40 and 50 F-box genes. Less than 40 genes were found in Chr6 (29, 5.29%), Chr16 (24, 4.38%), Chr14 (23, 4.20%), and Chr2 (18, 3.28%). The uneven chromosomal distribution of F-box genes may imply distinct expression patterns, as chromatin environments vary across chromosomal regions and can influence gene expression regulation.

**Figure 5:**
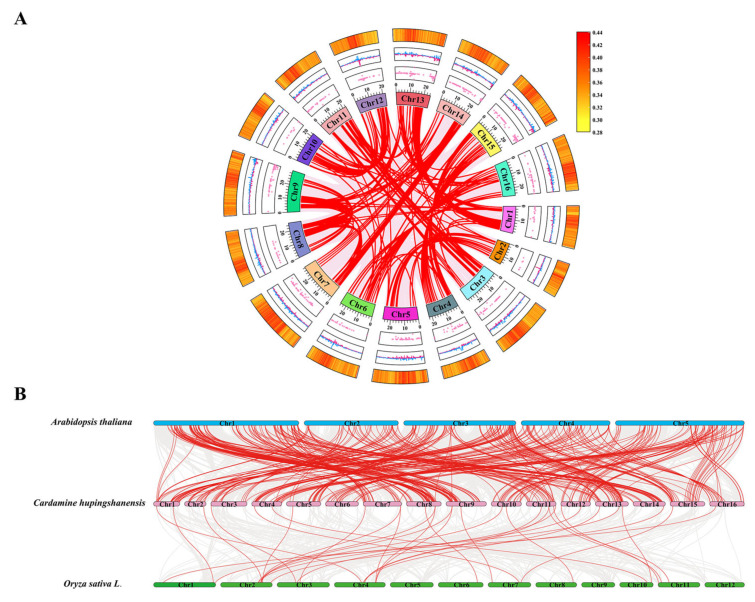
Collinearity analysis of F-box genes in *C. hupingshanensis.* (**A**) Circle figure of the duplicated gene pairs of the *ChFBX* genes. The red lines represent the duplicated gene pairs of *ChFBX* genes. (**B**) Synteny analysis of F-box genes between *C. hupingshanensis*, *A. thaliana* and *O. sativa*. The red lines represent the syntenic F-box pairs. The gray lines indicate genome-wide background collinearity, and the red lines represent the connecting lines of homologous duplicated genes of the target gene family.

Gene segmental duplication is considered a major driver of evolutionary processes and an important mechanism underlying the emergence of biological diversity [28,29]. It provides organisms with a rich source of genetic material, creating ample opportunity for natural selection to act upon and ultimately drive the emergence of novel biological features and capabilities [30,31]. To investigate the presence of gene duplication events among the F-box genes in *C. hupingshanensis*, segmental duplication analysis was performed. As shown in Figure 5A, a total of 283 collinear gene pairs were identified on different chromosomes, indicating segmental duplication events (Table S4). These findings show that segmental duplication may play an important role in the expansion of the F-box family genes in *C. hupingshanensis*. In addition, the collinearity of F-box genes between *C. hupingshanensis*, *A. thaliana* and *O. sativa* was comparatively analyzed (Figure 5B). The results showed that a total of 330 *ChFBX* genes were collinear with 225 *A. thaliana* genes and 408 gene pairs were detected (Table S5). In contrast, only 27 genes in *C. hupingshanensis* exhibited collinearity with 20 rice genes, comprising 31 gene pairs. These results suggested a relatively closer evolutionary relationship between *C. hupingshanensis* and Arabidopsis compared to rice, further supporting the similarity in genomic structure among Brassicaceae plants.

### 3.4. Cis-Acting Elements in the Promoters of ChFBX Genes

**Figure 6:**
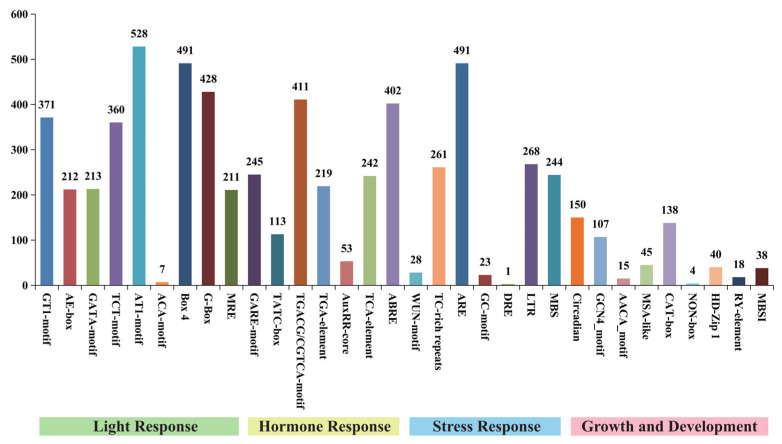
Cis-elements identified in promoters of the 548 F-box genes in *C. hupingshanensis*. The cis-element types and the numbers identified in the promoters of *ChFBX* genes in *C. hupingshanensis* are marked in the figure.

*Cis*-acting elements located in the promoter regions of encoding genes play a critical role in regulating gene transcriptional expression [32]. To analyze the *cis*-acting elements in the promoters of F-box genes and the functions of these genes, the 2000 bp putative promoter sequences of the 548 F-box genes were uploaded to the online database PlantCARE. The results presented 32 distinct *cis*-elements which were divided into four groups: light responsiveness, including nine types of elements; hormone responsiveness, including seven types of elements; stress responsiveness, including seven types of elements; and growth and development, including nine types of elements (Figure 6 and Figure S2 and Table S6). Notably, cis-elements such as the AT1 motif, Box 4, the G-Box, the TGACG motif, ABRE and ARE exist abundantly in F-box genes, suggesting their important role in light response, MeJA and ABA regulation, and other stress signal transduction regulation processes. Taken together, F-box genes in *C. hupingshanensis* are involved in several processes of plant growth, development and environment responses, suggesting the involvement of these genes in the regulation of such processes.

### 3.5. GO and KEGG Enrichment Analyses and Functional Prediction

**Figure 7:**
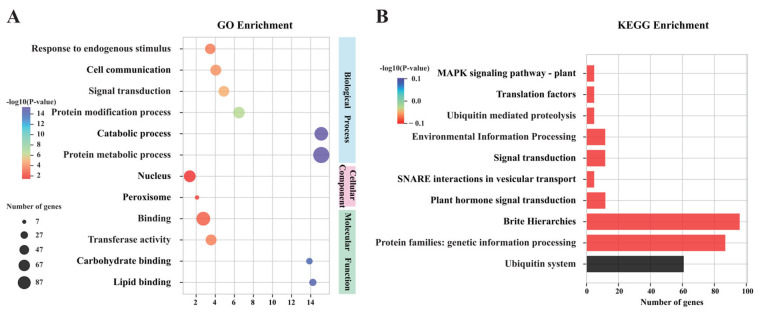
GO and KEGG pathway enrichment analyses of *ChFBX* genes. (**A**) GO enrichment analysis; (**B**) KEGG pathway enrichment analysis.

To elucidate the functional roles of the 548 F-box genes, Gene Ontology (GO) clustering analysis was conducted (Figure 7A). The clustering results provided significant insights into the molecular functions, cellular components, and biological processes associated with these genes. In the molecular function category, 47 and 76 F-box genes were predicted to be involved in transferase activity (GO:0016740) and binding (GO:0005488), respectively. This indicates that F-box proteins may play important roles in substrate recognition, protein–protein interactions, and ubiquitination processes, thereby participating in the fine regulation of various cellular activities. In the cellular component category, 55 F-box genes were annotated to the nucleus (GO:0005634), followed by some annotated to the peroxisome (GO:0005777). In the biological process category, 104, 74, 53, 47, 45, and 43 F-box genes were predicted to be involved in the protein metabolic process (GO:0019538), the catabolic process (GO:0009056), the protein modification process (GO:0036211), cell communication (GO:0007154), signal transduction (GO:0007165), and responses to an endogenous stimulus (GO:0009719), respectively.

To evaluate the KEGG pathways involved in the *ChFBX* genes, this study conducted KEGG enrichment analysis on the *ChFBX* genes. As shown in Figure 7B, *ChFBX* genes were significantly enriched in 10 KEGG pathways (*p* < 0.05), indicating their diverse regulatory functions. Among them, the ubiquitin system, ubiquitin-mediated proteolysis, and proteasome-related pathways showed the highest enrichment scores, which is consistent with the classical role of F-box proteins as substrate recognition subunits of SCF E3 ubiquitin ligase complexes. Additionally, pathways related to environmental information processing, plant hormone signal transduction, and MAPK signaling were also significantly enriched, suggesting that *ChFBX* genes may be involved in stress perception and hormone regulation. The presence of SNARE interactions in vesicular transport and protein processing in the endoplasmic reticulum further indicates that *ChFBX* genes may be involved in protein modification and intracellular transport. These results are consistent with the subsequent research conclusion that *ChFBX* proteins may regulate the abiotic stress response of *C. hupingshanensis* by mediating the degradation of target proteins. The above analysis results reveal the conserved and specific functional characteristics of *ChFBX* genes in *C. hupingshanensis*. In summary, these statistically significant enriched pathways not only reveal the conserved function of *ChFBX* genes in ubiquitin-mediated proteolysis but also demonstrate their species-specific participation in signal transduction and environmental adaptation.

### 3.6. Tissue Expression Analysis of Differentially Expressed ChFBX Genes Under Selenium Treatment

**Figure 8:**
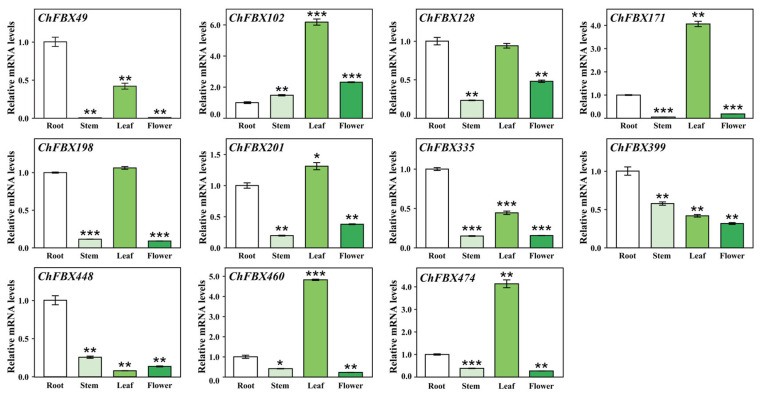
Tissue-specific expression of 11 *ChFBX* genes. Root, stem, leaf and flower tissues from *C. hupingshanensis* were collected for RNA extraction and tissue-specific expression detection of *ChFBX* genes. Significance was determined by Student’s *t*-test. * *p* < 0.05, ** *p* < 0.01, and *** *p* < 0.001.

Gene expression profiles provide valuable information for determining the biological functions of genes. To identify *ChFBX* genes responsive to exogenous selenium, we analyzed the transcriptome data of *C. hupingshanensis* under selenium treatment. A total of 11 differentially expressed genes were screened (Figure S3), and their expression patterns were investigated across various tissues including roots, stems, leaves, and flowers (Figure 8). qRT-PCR analysis revealed that *ChFBX102*, *ChFBX171*, *ChFBX460*, and *ChFBX474* exhibited the highest expression levels in leaves. Notably, *ChFBX102* also showed higher expression in stems and flowers compared to roots, suggesting that these genes may play regulatory roles in aerial tissues. Additionally, *ChFBX198* and *ChFBX201* displayed comparable expression levels in both roots and leaves, implying their potential functions in both organs. Since all these genes were responsive to exogenous selenium, they may be involved in regulating selenium uptake and allocation in different tissues, although further experimental validation is required.

### 3.7. Expression Profiles of ChFBX Genes in Response to Selenium as Well as Other Abiotic Stress Factors

**Figure 9:**
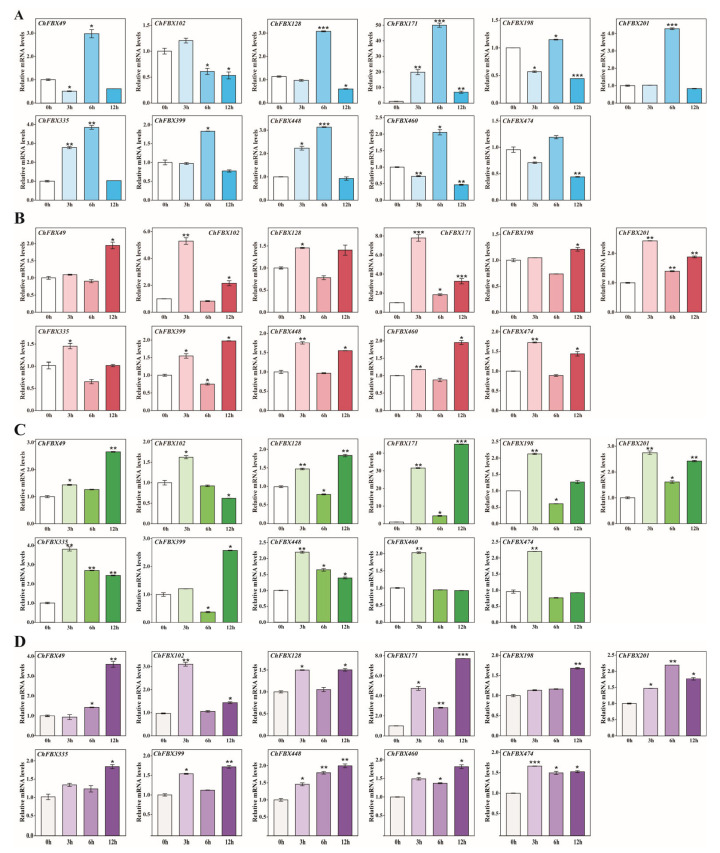
Expression profiles of 11 representative *ChFBX* genes in response to sodium selenate. Two-month-old seedlings of *C. hupingshanensis* were treated with 0.25 mg/L (**A**,**B**) and 16 mg/L (**C**,**D**) Na_2_SeO_4_ for 0, 3 h, 6 h, 12 h and the total RNA in leaves and roots was extracted severally for qRT-PCR. Significance was determined by Student’s *t*-test. * *p* < 0.05, ** *p* < 0.01, and *** *p* < 0.001.

As *C. hupingshanensis* possesses a characteristic of selenium hyperaccumulation, to investigate the expression response of F-box genes to exogenous selenium at the whole transcriptome level, the RNA-seq data analysis identified 11 differentially expressed F-box genes under both low-concentration (0.25 mg/L) and high-concentration (16 mg/L) treatments of Na_2_SeO_4_. The *C. hupingshanensis* seedlings which had been growing normally for 2 months were treated with Na_2_SeO_4_ at a low concentration of 0.25 mg/L and a high concentration 16 mg/L for 3 h, 6 h, and 12 h, respectively, to investigate the transient response of these 11 genes to exogenous selenium, and qRT-PCR analysis on the expression of these 11 genes was then performed (Figure 9). Under the 0.25 mg/L Na_2_SeO_4_ treatment, these genes exhibited distinct expression patterns in roots and leaves. In roots, distinct expression kinetics were observed. *ChFBX335* and *ChFBX448* showed transient upregulation. *ChFBX201* and *ChFBX399* were induced only at 6 h, with transcript levels increasing by approximately 4-fold and 1.9-fold, respectively. *ChFBX102* was downregulated at 6 and 12 h, decreasing to about 0.5-fold of the control. *ChFBX128* increased by 3-fold at 6 h and then decreased to 0.5-fold at 12 h. *ChFBX49*, *ChFBX198*, *ChFBX460*, and *ChFBX474* followed a triphasic pattern characterized by downregulation at 3 h, upregulation at 6 h, and downregulation again at 12 h. Among these, *ChFBX49* displayed the strongest changes, decreasing to 0.5-fold at 3 h and increasing to 3-fold at 6 h. Notably, *ChFBX171* exhibited the most pronounced induction, reaching approximately 50-fold higher than the control at 6 h. In leaves, *ChFBX171* also exhibited sustained upregulation, peaking at approximately 8-fold at 3 h, though this induction was lower than that in roots (approximately 50-fold at 6 h). For other genes, *ChFBX49*, *ChFBX198*, and *ChFBX460* showed delayed induction (2-fold, 1.5-fold, and 2-fold at 12 h); *ChFBX102*, *ChFBX335*, and *ChFBX474* peaked at 3 h (5-fold, 2-fold, and 2-fold) then attenuated; *ChFBX128*, *ChFBX201*, and *ChFBX448* displayed biphasic peaks at 3 h and 12 h (3-fold, 2-fold, and 2-fold) with a trough at 6 h; and *ChFBX399* decreased to 0.6-fold at 6 h then rebounded to 2-fold at 12 h.

When treated with 16 mg/L Na_2_SeO_4_, these genes exhibited markedly reprogrammed expression patterns in both roots and leaves. In roots, divergent expression kinetics were observed. *ChFBX102*, *ChFBX198*, *ChFBX460*, and *ChFBX474* showed transient early upregulation, peaking at 3 h (2- to 2.5-fold), followed by rapid attenuation. *ChFBX201*, *ChFBX335*, and *ChFBX448* also peaked at 3 h (2.5- to 3-fold) and remained above the baseline at 12 h. *ChFBX49*, *ChFBX128*, and *ChFBX399* exhibited sustained or delayed induction, progressively increasing to 2- to 3.5-fold at 12 h; notably, *ChFBX399* showed a sharp dip at 6 h before a strong rebound. *ChFBX171* displayed the most pronounced and sustained amplification, reaching 15-fold at 3 h, 30-fold at 6 h, and 55-fold at 12 h, with no sign of attenuation. In leaves, the expression patterns converged toward delayed activation. *ChFBX49*, *ChFBX128*, *ChFBX171*, *ChFBX198*, *ChFBX335*, *ChFBX399*, and *ChFBX448* all showed progressive or sustained upregulation, peaking at 12 h (ranging from 2- to 7.5-fold). *ChFBX460* and *ChFBX474* peaked at 6 h (approximately 2-fold and 1.5-fold, respectively). *ChFBX102* and *ChFBX201* displayed transient early induction at 3 h (approximately 2.5-fold and 1.5-fold, respectively), followed by attenuation, with *ChFBX201* showing a mild rebound at 12 h. *ChFBX171* again showed the strongest induction in leaves (approximately 7.5-fold at 12 h), though its magnitude was considerably lower than that observed in roots (55-fold). Overall, the leaf response exhibited reduced kinetic heterogeneity compared with that under the 0.25 mg/L treatment. These results demonstrate that a subset of F-box genes indeed responds to exogenous selenium treatment, and this response is tissue-specific.

**Figure 10:**
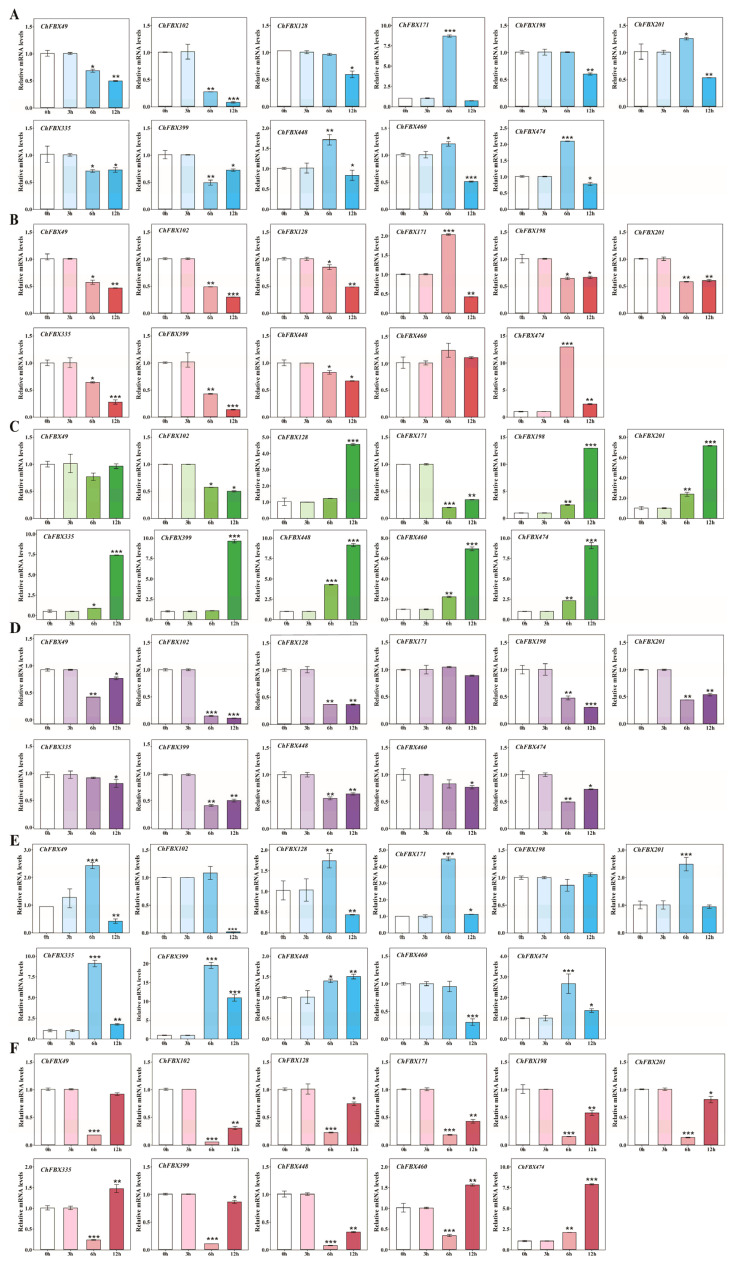
Relative expression level of 11 representative ChFBX genes under NaCl, PEG6000 and ABA treatment. Seedlings of *C. hupingshanensis* with 6–7 true leaves were treated with 150 mM NaCl, 20% PEG6000, and 50 μM ABA for 3, 6, and 12 h. The total RNA of 11 representative *ChFBX* genes was extracted separately from roots and leaves, reverse-transcribed, and then subjected to quantitative real-time PCR analysis. (**A**,**C**,**E**) Relative expression level of 11 representative ChFBX genes under NaCl, PEG6000 and ABA treatment in leaves. (**B**,**D**,**F**) Relative expression level of 11 representative ChFBX genes under NaCl, PEG6000 and ABA treatment in roots. Significance was determined by Student’s *t*-test. * *p* < 0.05, ** *p* < 0.01, and *** *p* < 0.001.

Under salt stress treatment (Figure 10A,B), most genes exhibited minimal changes in transcript abundance at 3 hpt in leaves. However, at 6 and 12 hpt, the expression levels of *ChFBX102* were significantly downregulated, decreasing to approximately 0.3-fold and 0.2-fold of the control, respectively. *ChFBX335* and *ChFBX399* were downregulated at 6 hpt (approximately 0.7-fold and 0.6-fold) and further reduced at 12 hpt (approximately 0.2-fold and 0.3-fold). *ChFBX49* showed no significant change at 3 and 6 hpt but was markedly reduced at 12 hpt (0.4-fold). In contrast, *ChFBX128* and *ChFBX198* showed no significant alterations at 3 and 6 hpt but were markedly reduced at 12 hpt (approximately 0.6-fold and 0.7-fold). The remaining genes, including *ChFBX171*, *ChFBX201*, *ChFBX448*, *ChFBX460*, and *ChFBX474*, exhibited a transient induction followed by a decline, peaking at 6 hpt (approximately 9-fold, 3-fold, 2-fold, 2-fold, and 4-fold, respectively). Among these, *ChFBX171* displayed the most pronounced upregulation, with transcript levels reaching approximately 9-fold higher than those of the control at 6 hpt. In roots, the expression patterns of most genes were similar to those observed in leaves, with progressive downregulation at 6 and 12 hpt. Notably, *ChFBX171* and *ChFBX474* were significantly upregulated at 6 h post-treatment, reaching approximately 1.5-fold and 12-fold, respectively, with *ChFBX474* exhibiting a more than tenfold increase in transcript abundance. To simulate osmotic stress, seedlings were treated with 20% PEG 6000. Under this condition, most genes exhibited minimal changes in transcript abundance at 3 hpt in leaves. However, at 6 and 12 hpt, the expression levels of *ChFBX102* were significantly downregulated, decreasing to approximately 0.7-fold and 0.5-fold of the control, respectively. *ChFBX460* was downregulated at 6 hpt (0.7-fold) and further reduced at 12 hpt (0.5-fold). *ChFBX49* and *ChFBX198* showed no significant change at 3 and 6 hpt but were markedly reduced at 12 hpt (0.5-fold and 0.6-fold). In contrast, *ChFBX128* and *ChFBX171* showed no significant alterations at 3 and 6 hpt but were markedly reduced at 12 hpt (0.5-fold and 0.6-fold). The remaining genes, including *ChFBX201*, *ChFBX335*, *ChFBX399*, *ChFBX448*, and *ChFBX474*, exhibited sustained upregulation, reaching maximum levels at 12 hpt (6-fold, 8-fold, 8-fold, 8-fold, and 8-fold, respectively). Among these, *ChFBX335*, *ChFBX399*, *ChFBX448*, and *ChFBX474* displayed the most pronounced upregulation, with transcript levels reaching approximately 8-fold higher than those of the control at 12 hpt (Figure 10C). In roots, the expression patterns of most genes were similar to those observed in leaves, with progressive downregulation at 6 and 12 hpt. Notably, *ChFBX474* was uniquely upregulated at 12 h post-treatment, reaching approximately 2-fold, while *ChFBX128* and *ChFBX201* exhibited the most pronounced downregulation, decreasing to approximately 0.2-fold at 12 hpt (Figure 10D).

As a key phytohormone regulating abiotic stress responses, abscisic acid (ABA) plays a central role in plant adaptation to adverse environments. To explore the transcriptional responses of F-box genes to ABA signaling, *C. hupingshanensis* seedlings were subjected to exogenous ABA treatment, and the expression dynamics of selected F-box genes were examined in both leaf and root tissues. In leaves (Figure 10E), most genes exhibited minimal changes in transcript abundance at 3 hpt. However, at 6 and 12 hpt, the expression levels of *ChFBX102* were significantly downregulated, decreasing to approximately 0.7-fold and 0.5-fold of the control, respectively. *ChFBX460* was downregulated at 6 hpt (0.7-fold) and further reduced at 12 hpt (0.5-fold). *ChFBX49* showed no significant change at 3 and 6 hpt but was markedly reduced at 12 hpt (0.5-fold). In contrast, *ChFBX128* and *ChFBX198* showed no significant alterations at 3 and 6 hpt but were markedly reduced at 12 hpt (0.6-fold and 0.7-fold). The remaining genes, including *ChFBX171*, *ChFBX201*, *ChFBX335*, *ChFBX399*, *ChFBX448*, and *ChFBX474*, exhibited a transient induction followed by a decline, peaking at 6 hpt (5-fold, 3-fold, 9-fold, 9-fold, 2-fold, and 4-fold, respectively). Among these, *ChFBX335* and *ChFBX399* displayed the most pronounced upregulation, with transcript levels reaching approximately 9-fold higher than those of the control at 6 hpt. In roots (Figure 10F), the expression patterns diverged from those observed in leaves. *ChFBX102*, *ChFBX128*, *ChFBX171*, *ChFBX198*, and *ChFBX201* exhibited progressive downregulation at 6 and 12 hpt, with *ChFBX171* and *ChFBX198* decreasing to approximately 0.1-fold at 12 hpt. In contrast, *ChFBX49*, *ChFBX335*, *ChFBX399*, *ChFBX448*, and *ChFBX460* showed delayed upregulation, reaching maximum levels at 12 hpt (1.5-fold, 2.0-fold, 1.5-fold, 1.5-fold, and 2.0-fold, respectively). Notably, *ChFBX474* was significantly upregulated at 6 h post-treatment, reaching approximately 2.5-fold.

Collectively, these results demonstrate that distinct F-box genes exhibit temporally and spatially dynamic expression patterns in response to different abiotic stresses, including selenium, salt, osmotic stress, and ABA. This transcriptional reprogramming of the F-box genes suggests their potential involvement in stress adaptation, providing a foundation for future functional studies to investigate their molecular roles.

### 3.8. Plants Overexpressing ChFBX171 Exhibit Heightened Sensitivity to Salt Stress During Seed Germination

**Figure 11:**
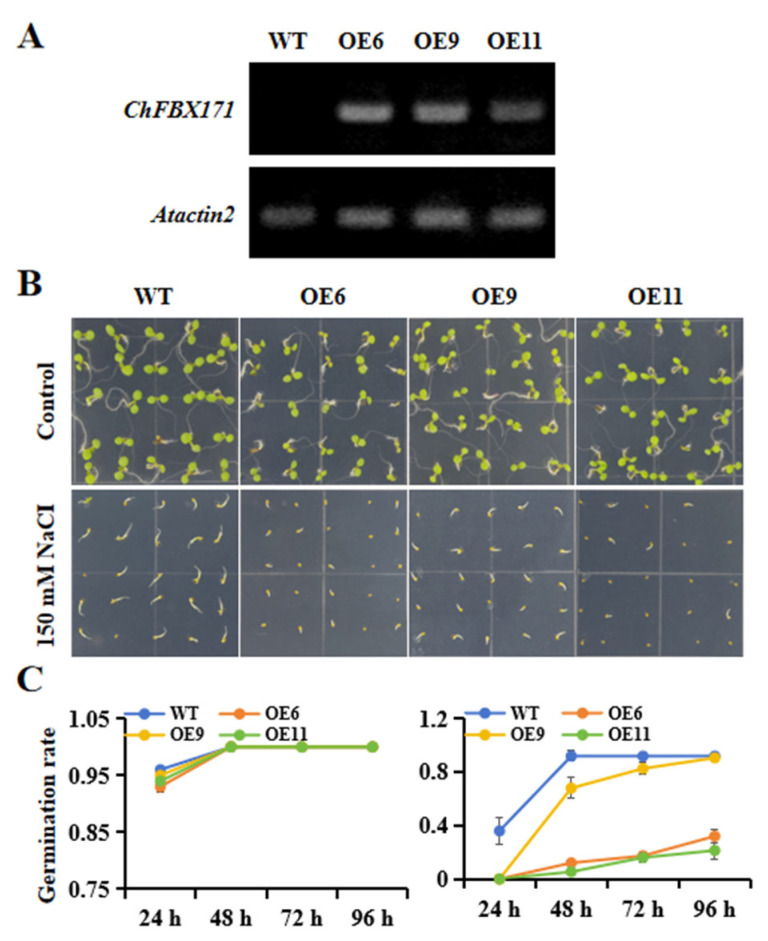
Semi-quantitative RT-PCR analysis of *ChFBX171* expression in transgenic lines and the performance of WT and ChFBX171 OE plants under salt stress. (**A**) Semi-quantitative RT-PCR analysis of *ChFBX171* expression in wild-type (WT) and three independent overexpression lines (OE6, OE9, and OE11). *AtActin2* was used as an internal control. The PCR cycles were 28 for *ChFBX171* and 25 for *AtActin2*. (**B**) The phenotype of WT and ChFBX171 OE plants under control and salt stress. (**C**) The germination rate of WT and ChFBX171 OE plants under control (left) and salt stress (right). The original blot images for (**A**) are provided in the Supplementary Materials (File S1).

To investigate the function of the *ChFBX* genes, we selected the *ChFBX171* gene, which exhibits a strong response to various abiotic stresses. For further study, we constructed an overexpression vector of this gene and heterologously transformed it into wild-type *Arabidopsis thaliana* Col-0 using the Agrobacterium-mediated method. Subsequent semi-quantitative RT-PCR analysis confirmed the overexpression of *ChFBX171* in multiple independent lines, among which three lines (OE6, OE9, and OE11) with relatively higher expression levels were selected for further experiments (Figure 11A).

Unexpectedly, when treated with NaCl, we observed that the germination of OE6, OE9, and OE11 was significantly slower than that of the WT (Figure 11), and their germination rates were also markedly lower. Specifically, under controlled conditions (without NaCl stress), no significant differences in seed germination rates were observed among the wild-type (WT) and three independent *ChFBX171* overexpression lines (OE6, OE9, and OE11). However, under the 150 mM NaCl treatment, all three transgenic lines exhibited reduced germination rates compared to the WT at different time points, although the degree of inhibition varied among lines. These results indicate that heterologous overexpression of *ChFBX171* in Arabidopsis leads to increased sensitivity to salt stress, especially during the seed germination stage, suggesting that *ChFBX171* may play a role in salt stress responses. However, these findings were obtained in a heterologous system, and the native function of *ChFBX171* in *C. hupingshanensis* remains to be further investigated.

## 4. Discussion

*C. hupingshanensis* is a selenium-hyperaccumulating plant species endemic to China; is particularly abundant in the selenium-rich region of Enshi, Hubei Province; and has been recognized as the “king of selenium-enriched plants.” This species is valued not only for its palatable taste and high nutritional content but also for its suitability for large-scale cultivation and processing. Its commercial production increases dietary selenium intake for consumers and generates significant economic returns for farmers, thereby promoting regional agricultural restructuring and delivering substantial socioeconomic benefits. Moreover, owing to its exceptional selenium accumulation capacity, *C. hupingshanensis* has been adopted as a novel selenium source in various applications, including the livestock industry and functional food production [33]. These unique characteristics make *C. hupingshanensis* an ideal model for studying the molecular mechanisms of selenium metabolism and for developing selenium-biofortified products.

The ubiquitin–proteasome system is a major post-translational regulatory mechanism in plants. In this system, the E3 ubiquitin ligase is the key component that determines substrate specificity. This specificity is achieved through the specific binding of F-box proteins to their substrates. As the substrate-recognition subunits of the Skp1–Cullin–F-box (SCF) E3 ubiquitin ligase complex, F-box proteins recruit substrates to the SCF complex, targeting them for degradation by the 26S proteasome. Due to the diversity of F-box substrates, the number of F-box gene family members is relatively larger in different species compared to other gene families. In fact, in contrast to mammals like mice (74) and humans (68) [34,35], higher plants possess a significantly greater number of F-box proteins in their genomes. For example, *Medicago truncatula* contains 972 F-box genes, the highest number reported in any plant species so far [21]. In the model plant *Arabidopsis thaliana* and the crop species rice (*Oryza sativa*), 694 and 687 F-box genes were identified [15,16]. In contrast, the tomato (*Solanum lycopersicum*) has a considerably smaller F-box gene family, comprising 139 members [36]. In this study, a total of 548 F-box genes were identified based on the whole-genome files of *C. hupingshanensis*. These *ChFBX* genes are distributed relatively randomly across the 16 chromosomes of *C. hupingshanensis*. Although the number of F-box genes identified in *C. hupingshanensis* is lower than that in *Arabidopsis thaliana* and rice, this gene family has also experienced expansion over the course of evolution. Our analysis revealed 283 collinear gene pairs within *C. hupingshanensis*, implicating segmental duplication as a major driver of *ChFBX* family expansion. Furthermore, comparative synteny analysis demonstrated that *C. hupingshanensis* shares 408 homologous FBX pairs with *A. thaliana* but only 31 pairs with *O. sativa*, consistent with their closer phylogenetic relationship as Brassicaceae dicots.

Based on the GO and KEGG enrichment analyses, the potential roles of F-box genes in stress response can be reasonably linked to their predicted molecular functions and pathways. GO analysis indicated that F-box genes are significantly enriched in terms related to ubiquitin-dependent protein degradation, hormone signaling, and responses to an abiotic stimulus. This is generally consistent with the previously established functions of F-box proteins, which include ubiquitin-mediated protein degradation, regulation of ABA signaling, and participation in abiotic stress responses [37,38]. In addition, pathways related to abscisic acid (ABA) signaling and reactive oxygen species (ROS) homeostasis were also overrepresented. In fact, through Arabidopsis transgenesis, we also identified a *ChFBX171* gene that participates in the regulation of salt stress. Furthermore, the expression patterns of the 11 representative F-box genes showed varying degrees of responsiveness to exogenous abiotic stresses and ABA, with tissue-specific expression profiles. These results indicate that these F-box genes are transcriptionally responsive to stress conditions, which is consistent with previous reports showing that F-box genes are involved in salt stress regulation [39]. However, transcriptional responsiveness does not necessarily imply direct functional involvement. Further functional studies are required to determine whether and how these genes contribute to stress adaptation.

As a crucial component of post-transcriptional gene regulation, F-box proteins serve as substrate receptors for SCF E3 ubiquitin ligases, making them critical molecular switches that swiftly modulate the proteome in response to environmental signals. Substantial evidence showed that F-box genes are widely involved in the regulation of abiotic stresses in plants, such as drought and salt stress [40]. For instance, OsFBX257 in rice is involved in the regulation of drought stress and ABA signaling, while OsFBO13 is involved in the regulation of salt stress [41,42]. AtSDR in *Arabidopsis thaliana* is simultaneously involved in the regulation of both drought and salt stress [43]. In this study, we identified 548 F-box genes from *C. hupingshanensis* and selected 11 F-box genes from transcriptomic data for validation of their gene expression. The validation results showed that most of the selected F-box genes responded to selenium, NaCl, ABA, and PEG treatments, which is largely consistent with the findings from the promoter cis-element analysis. We then characterized the function of ChFBX171 and demonstrated its role in plant responses to salt stress. The findings align with a growing body of evidence implicating specific F-box family members as negative regulators of salt tolerance. For instance, in rice, the F-box protein MAIF1 (OsFBX325) negatively regulates salt tolerance by modulating ABA-mediated pathways and ROS scavenging [44]. The increased salt sensitivity observed in our *ChFBX171* overexpression lines, as evidenced by reduced germination rates, strongly suggests that ChFBX171 targets one or more positive regulators of stress tolerance or germination for proteasomal degradation. This mechanism would mirror that of other F-box proteins that fine-tune stress responses by eliminating proteins that promote growth or stress adaptation under optimal conditions, ensuring a rapid and resource-efficient shift in physiological states. The significant upregulation of ChFBX171 transcription under selenium, salt, osmotic, and ABA treatments suggests its involvement in a broad spectrum of abiotic stress signaling pathways. Notably, the most pronounced transcriptional induction was observed under salt stress, which correlated with our phenotypic analysis showing that ChFBX171 overexpression in *Arabidopsis* resulted in enhanced salt sensitivity during seed germination. The above findings represent preliminary results and indicate that ChFBX171 may positively regulate salt signals but negatively affect salt tolerance during seed germination in Arabidopsis. Nevertheless, the molecular mechanism through which *ChFBX171* mediates salt stress responses remains to be further elucidated.

## 5. Conclusions

In this study, a total of 548 F-box genes were identified from the genome of *C. hupingshanensis* and phylogenetically classified into nine distinct groups. These genes exhibited variable intron/exon structures and were unevenly distributed across 16 chromosomes. Collinearity analysis revealed 283 segmental duplication events, suggesting that gene family expansion has been primarily driven by segmental duplications. Promoter analysis identified 32 distinct cis-regulatory elements associated with stress and hormone responsiveness. The encoded F-box proteins displayed diverse physicochemical properties, subcellular localizations, and conserved domain architectures. Transcriptome profiling coupled with qRT-PCR validation uncovered 11 *ChFBX* genes responsive to exogenous selenium treatment. Notably, these genes were also differentially expressed under salt, osmotic stress, and abscisic acid (ABA) treatments. A representative gene, *ChFBX171*, was functionally characterized and was shown to negatively regulate salt stress tolerance during seed germination. Collectively, this comprehensive analysis of the F-box gene family in *C. hupingshanensis* provides a useful genomic resource and suggests potential associations of certain *ChFBX* genes with abiotic stress responses, particularly salt stress.

## Data Availability

The genome sequence reads of *C. hupingshanensis* are available at the NCBI Sequence Read Archive (SRA) as Bioproject PRJNA565347. The genome assembly sequences and gene annotations can be downloaded from the Genome Warehouse BIG Data Center under the accession number PRJCA002827. The RNA-seq datasets can be obtained from the National Center for Biotechnology Information using the accession number PRJNA590869. All other data are available within the article and its Supplementary Materials.

## Supplementary Materials

The following supporting information can be downloaded at https://www.mdpi.com/article/10.3390/biology15131003/s1, Figure S1: Statistical result of subcellular localization of ChFBX proteins in *C. hupingshanensis*. Figure S2: The cis-acting elements in the promoters of *ChFBX* genes in *C. hupingshanensis*. The cis-element types and the numbers identified in the promoters of *ChFBX* genes were marked in the figure. Figure S3: Transcriptome sequencing analysis result of 11 differentially expressed ChFBX genes in *C. hupingshanensis*. Table S1: The primer sequences used for qRT-PCR and semi-quantitative RT-PCR. Table S2: The list of 548 ChFBX genes in *C. hupingshanensis*. Table S3: The basic physicochemical properties and subcellular localization of 548 ChFBX proteins in *C. hupingshanensis.* Table S4: The duplicated gene pairs of the F-box gene family in *C. hupingshanensis*. Table S5: Synteny analysis of F-box genes between *C. hupingshanensis*, *A. thaliana* and *O. sativa*. Table S6: Cis-acting elements in the promoter of ChFBX genes in *C. hupingshanensis*. File S1: The original blot images for Figure 11A.

## Abbreviations

The following abbreviations are used in this manuscript: SCF: SKP1–Cullin–F-box; qRT-PCR: quantitative real-time PCR; kDa: kilodalton; pI: isoelectric point; GRAVY: grand average of hydropathy; FPKM: Fragments Per Kilobase of transcript per Million mapped reads; PCA: principal component analysis; DEG: differentially expressed gene; UTRs: untranslated regions; GO: Gene Ontology; KEGG: Kyoto Encyclopedia of Genes and Genomes; Me-JA: methyl jasmonate; ABA: abscisic acid; OE: overexpression; SOS: salt overly sensitive; ROS: reactive oxygen species.

## References

[1] Hershko A., Ciechanover A., Varshavsky A. (2000). The ubiquitin system. Nat. Med..

[2] Zheng N., Schulman B.A., Song L., Miller J.J., Jeffrey P.D., Wang P., Chu C., Koepp D.M., Elledge S.J., Pagano M. (2002). Structure of the Cul1–Rbx1–Skp1–F boxSkp2 SCF ubiquitin ligase complex. Nature.

[3] Cardozo T., Pagano M. (2004). The SCF ubiquitin ligase: Insights into a molecular machine. Nat. Rev. Mol. Cell Biol..

[4] de Roij M., Borst J.W., Weijers D. (2024). Protein degradation in auxin response. Plant Cell.

[5] Geem K.R., Kim H., Ryu H. (2022). SCF(FBS1) Regulates Root Quiescent Center Cell Division via Protein Degradation of APC/C(CCS52A2). Mol. Cells.

[6] Jurkiewicz P., Melser S., Maucourt M., Ayeb H., Veljanovski V., Maneta-Peyret L., Hooks M., Rolin D., Moreau P., Batoko H. (2018). The multistress-induced Translocator protein (TSPO) differentially modulates storage lipids metabolism in seeds and seedlings. Plant J..

[7] Liu J., Lin Q.F., Qi S.L., Feng X.J., Han H.L., Xu T., Hua X.J. (2020). The F-box protein EST1 modulates salt tolerance in *Arabidopsis* by regulating plasma membrane Na+/H+ antiport activity. J. Plant Physiol..

[8] Jia F., Wang C., Huang J., Yang G., Wu C., Zheng C. (2015). SCF E3 ligase PP2-B11 plays a positive role in response to salt stress in *Arabidopsis*. J. Exp. Bot..

[9] Gao L., Jia S., Cao L., Ma Y., Wang J., Lan D., Guo G., Chai J., Bi C. (2022). An F-box protein from wheat, TaFBA-2A, negatively regulates JA biosynthesis and confers improved salt tolerance and increased JA responsiveness to transgenic rice plants. Plant Physiol. Biochem..

[10] Zhang X., Sun R., Zhan F., Yang G., Wang Y., Yu Y., Ni Z. (2025). The Gma-miR4359b/GmFBX193 module is involved in the response of soybean to salt stress. Plant Sci..

[11] Yu L., Liu W., Guo Z., Li Z., Jiang H., Zou Q., Mao Z., Fang H., Zhang Z., Wang N. (2020). Interaction between MdMYB63 and MdERF106 enhances salt tolerance in apple by mediating Na^+^/H^+^ transport. Plant Physiol. Biochem..

[12] Devate N.B., Krishna H., Sunilkumar V.P., Manjunath K.K., Mishra C.N., Jain N., Singh G.P., Singh P.K. (2022). Identification of genomic regions of wheat associated with grain Fe and Zn content under drought and heat stress using genome-wide association study. Front. Genet..

[13] Fang Q., Zhou F., Zhang Y., Singh S., Huang C.F. (2021). Degradation of STOP1 mediated by the F-box proteins RAH1 and RAE1 balances aluminum resistance and plant growth in *Arabidopsis thaliana*. Plant J..

[14] Yang Y., Wang R., Wang L., Cui R., Zhang H., Che Z., Hu D., Chu S., Jiao Y., Yu D. (2023). GmEIL4 enhances soybean (*Glycine max*) phosphorus efficiency by improving root system development. Plant Cell Environ..

[15] Xu G., Ma H., Nei M., Kong H. (2009). Evolution of F-box genes in plants: Different modes of sequence divergence and their relationships with functional diversification. Proc. Natl. Acad. Sci. USA.

[16] Jain M., Nijhawan A., Arora R., Agarwal P., Ray S., Sharma P., Kapoor S., Tyagi A.K., Khurana J.P. (2007). F-Box Proteins in Rice. Genome-Wide Analysis, Classification, Temporal and Spatial Gene Expression during Panicle and Seed Development, and Regulation by Light and Abiotic Stress. Plant Physiol..

[17] Jia F., Wu B., Li H., Huang J., Zheng C. (2013). Genome-wide identification and characterisation of F-box family in maize. Mol. Genet. Genom..

[18] Jia Q., Xiao Z.X., Wong F.L., Sun S., Liang K.J., Lam H.M. (2017). Genome-Wide Analyses of the Soybean F-Box Gene Family in Response to Salt Stress. Int. J. Mol. Sci..

[19] Cui H.R., Zhang Z.R., Lv W., Xu J.N., Wang X.Y. (2015). Genome-wide characterization and analysis of F-box protein-encoding genes in the *Malus domestica* genome. Mol. Genet. Genom..

[20] Zhang S., Tian Z., Li H., Guo Y., Zhang Y., Roberts J.A., Zhang X., Miao Y. (2019). Genome-wide analysis and characterization of F-box gene family in *Gossypium hirsutum* L. BMC Genom..

[21] Song J.B., Wang Y.X., Li H.B., Li B.W., Zhou Z.S., Gao S., Yang Z.M. (2015). The F-box family genes as key elements in response to salt, heavy mental, and drought stresses in *Medicago truncatula*. Funct. Integr. Genom..

[22] Mo F., Zhang N., Qiu Y., Meng L., Cheng M., Liu J., Yao L., Lv R., Liu Y., Zhang Y. (2021). Molecular Characterization, Gene Evolution and Expression Analysis of the F-Box Gene Family in Tomato (*Solanum lycopersicum*). Genes.

[23] Yuan L., Zhu Y., Lin Z.Q., Banuelos G., Li W., Yin X. (2013). A novel selenocystine-accumulating plant in selenium-mine drainage area in Enshi, China. PLoS ONE.

[24] Cui L., Zhao J., Chen J., Zhang W., Gao Y., Li B., Li Y.F. (2018). Translocation and transformation of selenium in hyperaccumulator plant *Cardamine enshiensis* from Enshi, Hubei, China. Plant Soil.

[25] Huang C., Ying H., Yang X., Gao Y., Li T., Wu B., Ren M., Zhang Z., Ding J., Gao J. (2021). The *Cardamine enshiensis* genome reveals whole genome duplication and insight into selenium hyperaccumulation and tolerance. Cell Discov..

[26] Rao S., Yu T., Cong X., Xu F., Lai X., Zhang W., Liao Y., Cheng S. (2020). Integration analysis of PacBio SMRT- and Illumina RNA-seq reveals candidate genes and pathway involved in selenium metabolism in hyperaccumulator *Cardamine violifolia*. BMC Plant Biol..

[27] Kim T.K. (2015). T test as a parametric statistic. Korean J. Anesth..

[28] Flagel L.E., Wendel J.F. (2009). Gene duplication and evolutionary novelty in plants. New Phytol..

[29] Panchy N., Lehti-Shiu M., Shiu S.-H. (2016). Evolution of Gene Duplication in Plants. Plant Physiol..

[30] Kondrashov F.A. (2012). Gene duplication as a mechanism of genomic adaptation to a changing environment. Proc. R. Soc. B Biol. Sci..

[31] Machado T.B., Picorelli A.C.R., de Azevedo B.L., Aquino I.L.M.d., Queiroz V.F., Rodrigues R.A.L., Araújo J.P., Ullmann L.S., Santos T.M.d., Marques R.E. (2023). Gene duplication as a major force driving the genome expansion in some giant viruses. J. Virol..

[32] Chen L.Y., Xin Y., Wai C.M., Liu J., Ming R. (2020). The role of cis-elements in the evolution of crassulacean acid metabolism photosynthesis. Hortic. Res..

[33] Xu X., Wei Y., Zhang Y., Jing X., Cong X., Gao Q., Cheng S., Zhu Z., Zhu H., Zhao J. (2022). A new selenium source from Se-enriched *Cardamine violifolia* improves growth performance, anti-oxidative capacity and meat quality in broilers. Front. Nutr..

[34] Jin J., Cardozo T., Lovering R.C., Elledge S.J., Pagano M., Harper J.W. (2004). Systematic analysis and nomenclature of mammalian F-box proteins. Genes Dev..

[35] Kipreos E.T., Pagano M. (2000). The F-box protein family. Genome Biol..

[36] Su C., Cui J., Liu Y., Luan Y. (2022). Genome-wide identification and characterization of the tomato F-box associated (FBA) protein family and expression analysis of their responsiveness to Phytophthora infestans. Gene.

[37] Gong C., Yin X., Ye T., Liu X., Yu M., Dong T., Wu Y. (2022). The F-Box/DUF295 Brassiceae specific 2 is involved in ABA-inhibited seed germination and seedling growth in *Arabidopsis*. Plant Sci..

[38] Gonzalez L.E., Keller K., Chan K.X., Gessel M.M., Thines B.C. (2017). Transcriptome analysis uncovers *Arabidopsis* F-BOX STRESS INDUCED 1 as a regulator of jasmonic acid and abscisic acid stress gene expression. BMC Genom..

[39] Xu Y., Li L.X., Yu X.M., Liu D.Q. (2015). The Functions of F-box Protein in Plant Resistance to Stress. Plant Physiol. J..

[40] Zhang X., Gou M., Liu C.J.J.P.C. (2013). Arabidopsis Kelch Repeat F-Box Proteins Regulate Phenylpropanoid Biosynthesis via Controlling the Turnover of Phenylalanine Ammonia-Lyase. Plant Cell.

[41] Chen Y., Dan Z., Li S. (2024). GROWTH REGULATING FACTOR 7-mediated arbutin metabolism enhances rice salt tolerance. Plant Cell.

[42] Sharma E., Bhatnagar A., Bhaskar A., Majee S.M., Kieffer M., Kepinski S., Khurana P., Khurana J.P. (2023). Stress-induced F-Box protein-coding gene OsFBX257 modulates drought stress adaptations and ABA responses in rice. Plant Cell Environ..

[43] Li B.W., Gao S., Yang Z.M., Song J.B. (2022). The F-box E3 ubiquitin ligase AtSDR is involved in salt and drought stress responses in *Arabidopsis*. Gene.

[44] Yan Y.S., Chen X.Y., Yang K., Sun Z.X., Fu Y.P., Zhang Y.M., Fang R.X. (2011). Overexpression of an F-box Protein Gene Reduces Abiotic Stress Tolerance and Promotes Root Growth in Rice. Mol. Plant.

