# Supplementary material for: Genome-Wide Characterization of the F-Box Gene Family in Cardamine hupingshanensis and Functional Analysis of ChFBX171
Source: Biology (Basel). 2026 Jun 25;15(13):1003. doi: 10.3390/biology15131003 (PMC13360171; doi:10.3390/biology15131003)
Supplement: Supplementary file 1 [file biology-15-01003-s001.zip › Figure. S1. Statistical result of Subcellular localization of ChFBX proteins in C. hupingshanensis.pdf]

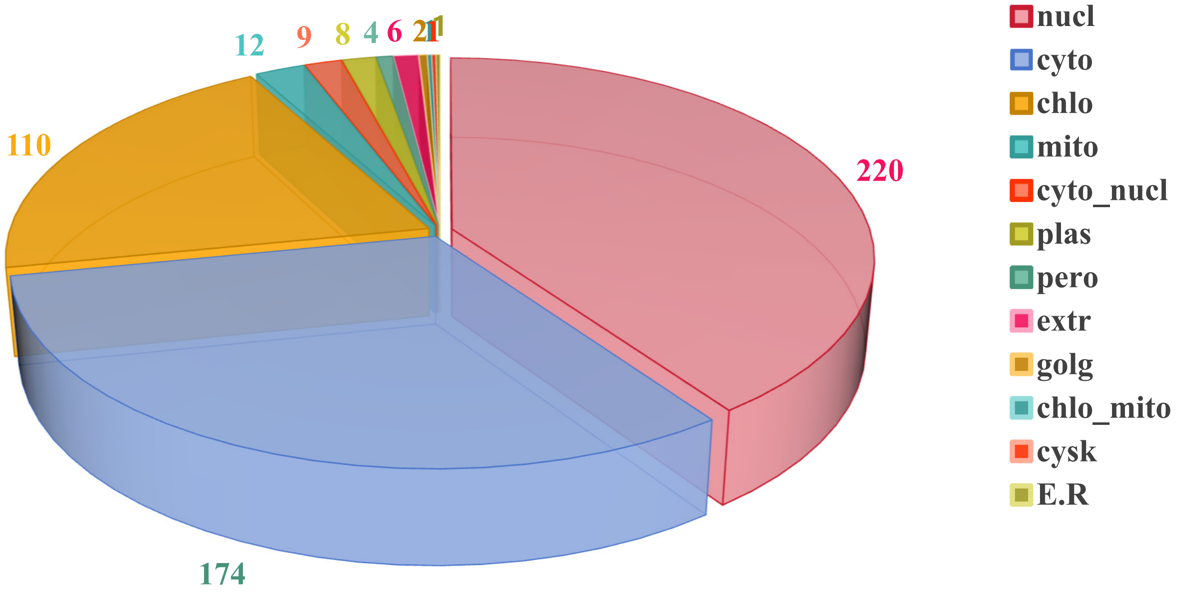

Figure S1. Statistical result of Subcellular localization of ChFBX proteins in *C. hupingshanensis*.
