# Supplementary material for: Genome-Wide Characterization of the F-Box Gene Family in Cardamine hupingshanensis and Functional Analysis of ChFBX171
Source: Biology (Basel). 2026 Jun 25;15(13):1003. doi: 10.3390/biology15131003 (PMC13360171; doi:10.3390/biology15131003)
Supplement: Supplementary file 1 [file biology-15-01003-s001.zip › File S1. The original uncropped blot images for Figure 11A.pdf]

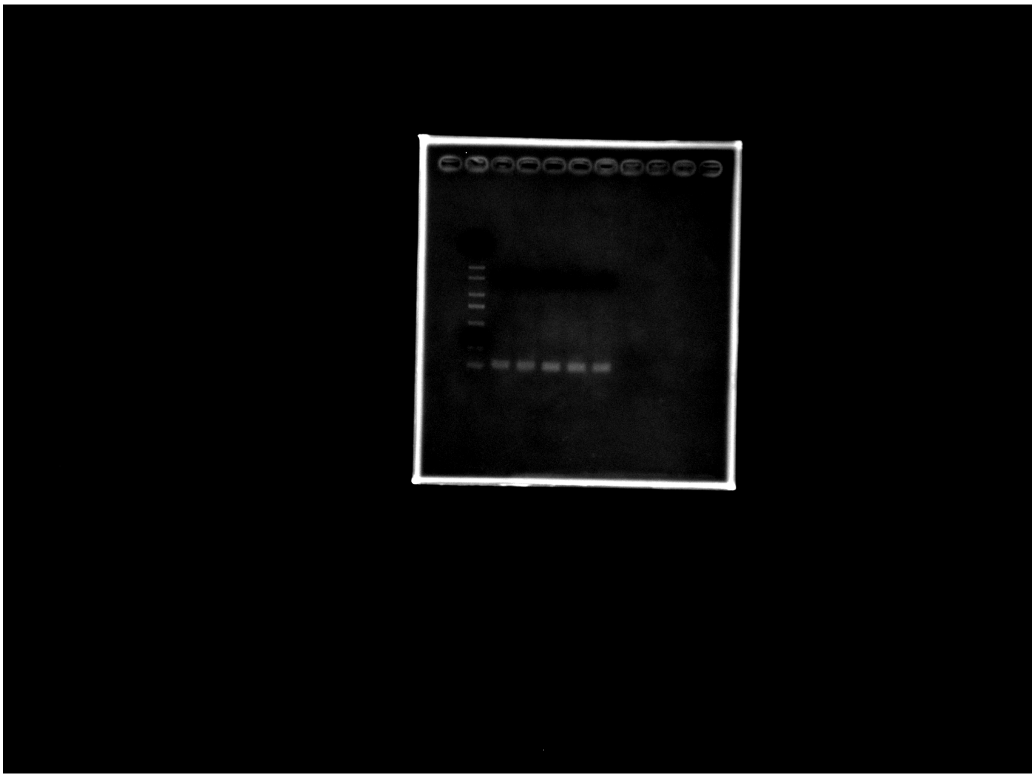

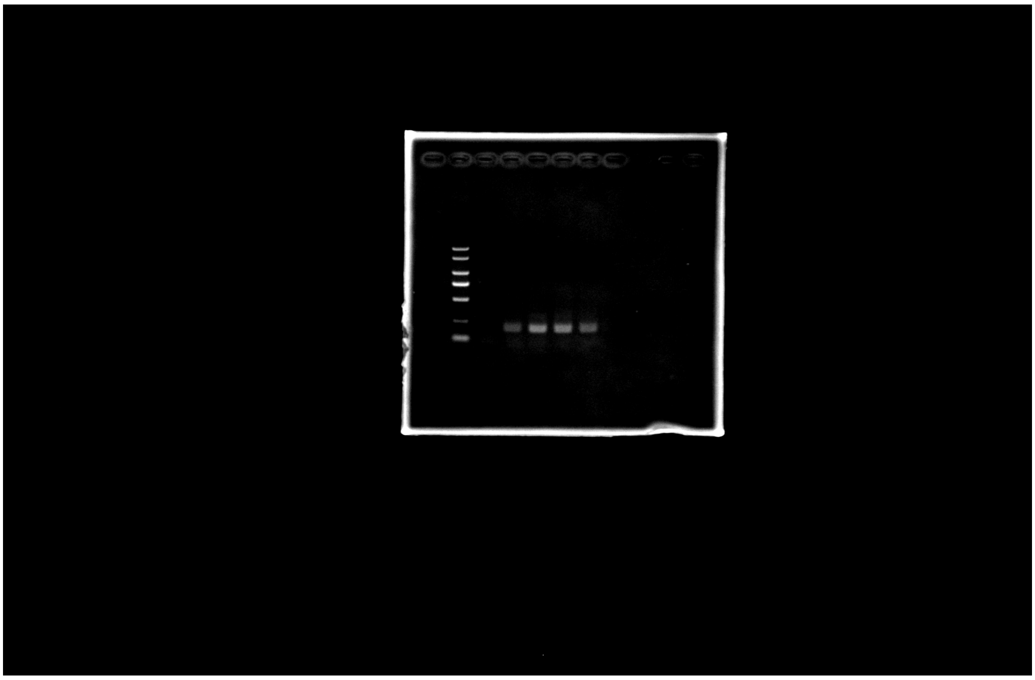

Following the DNA Maker (From bottom to top, the sizes are 100 bp, 250 bp, 500 bp, 750 bp, 1000 bp, 1500 bp, and 2000 bp.), lanes 1 to 5 correspond to WT, OE3, OE6, OE9, and OE11, respectively. The bands in the upper panel represent *AtActin2*, and those in the lower panel represent *ChFBX171*.
